# Supplementary figures and images for: Gender-based heterogeneity of FAHFAs in trained runners
Source: PLoS One. 2024 May 6;19(5):e0300037. doi: 10.1371/journal.pone.0300037 (PMC11073723; doi:10.1371/journal.pone.0300037)

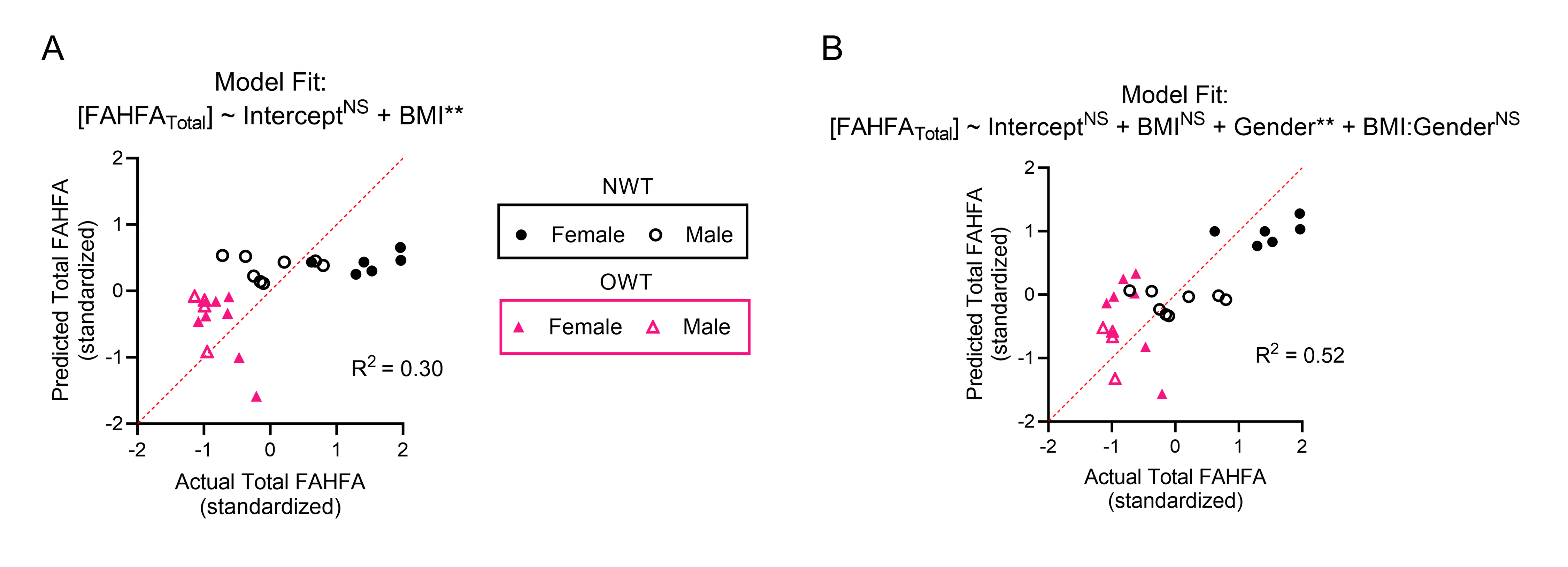

Supplement: S1 Fig — Actual standardized total FAHFA abundance versus predicted total circulating FAHFAs from regression model (~) (A) univariate effect of BMI, where the intercept indicates average circulating FAHFA for a participant with average BMI, and (B) effect of BMI adjusted for gender and the interaction between BMI: Gender, where intercept represents average FAHFA for male participant with average BMI. R2 value represents goodness of fit. **p<0.01, NS: not significant using One-way ANOVA with Tukey’s multiple comparisons test. (TIF) [file pone.0300037.s001.tif]

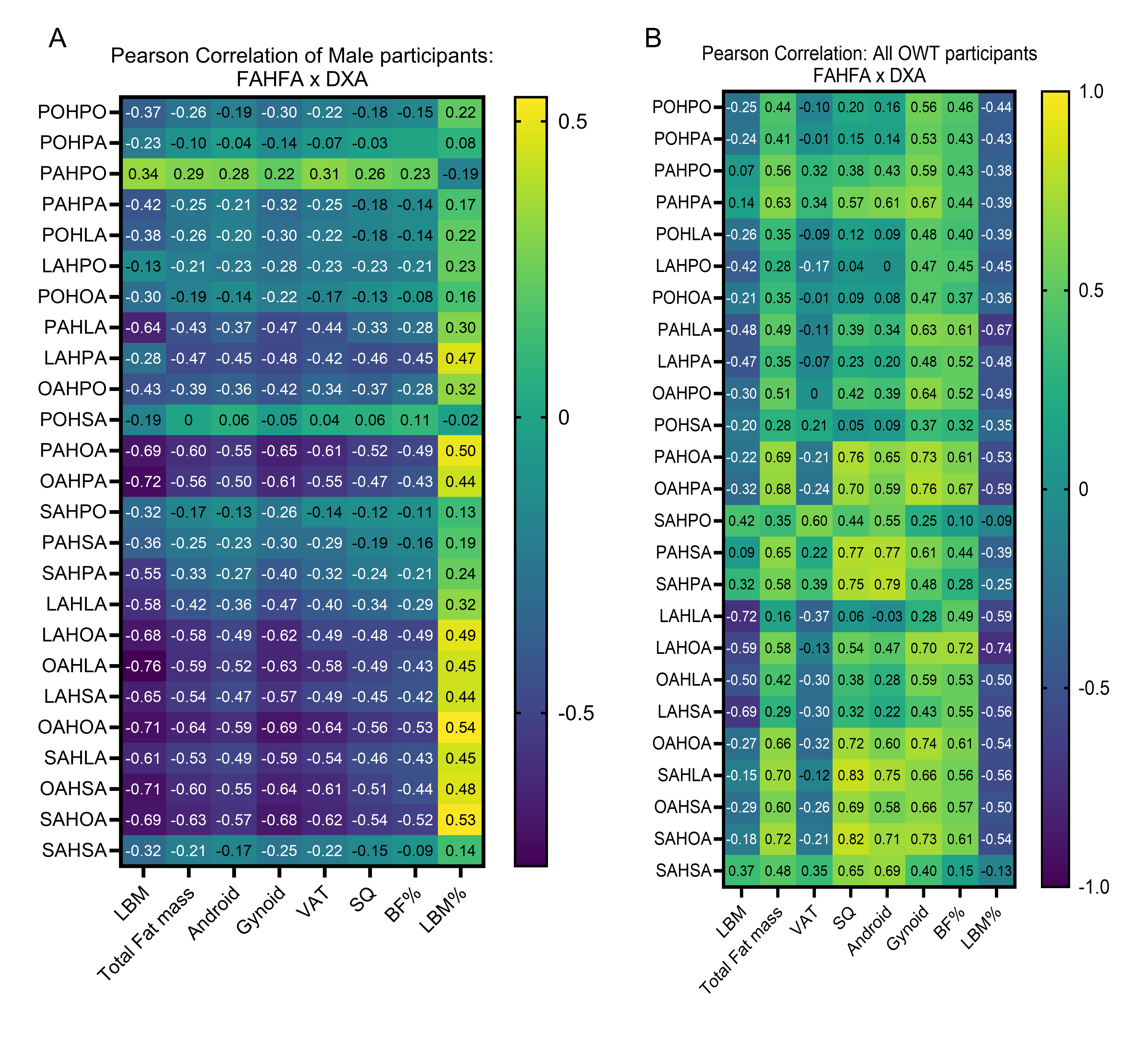

Supplement: S2 Fig — Heatmaps of Pearson correlation coefficients of FAHFAs with: (A) body composition in male participants; (B) body composition in all OWT participants. Labels indicate Pearson correlation coefficients (R). Species with an |R > 0.56| have an unadjusted p < 0.05, however none maintained significance after correction for multiple comparisons. (TIF) [file pone.0300037.s002.tif]
