# Supplementary material for: Gender-based heterogeneity of FAHFAs in trained runners
Source: PLoS One. 2024 May 6;19(5):e0300037. doi: 10.1371/journal.pone.0300037 (PMC11073723; doi:10.1371/journal.pone.0300037)
Supplement: Attachment — Submitted filename: Response_to_reviewers.docx [file pone.0300037.s004.docx]

**Responses to Reviewers**

**Reviewer #1:** The study entitled” Gender-based heterogeneity of FAHFAs in trained runner “investigated the relationship between baseline circulating FAHFAs in individuals who were self-reported runners relative to various body composition measurements.

The interpretation of the results was often confusing and needs to be rewritten. A few specific examples can be found below.

1. It is uncertain why “trained runners” is used in the title. How being aerobically trained may have influenced the results was not mentioned in the results or discussion. In addition, from the data presented, training appeared to have no effects on the study outcomes.

The investigators provide no evidence that their subjects were in fact aerobically trained. Participants were selected based solely on self-reported data about the number of times each week they participated in aerobic exercise; three times per week was the minimum inclusion criteria. In addition, the investigators said that participants were able to run for 90 minutes, but no information on this was provided. Without this information or any quantifiable fitness data, it is difficult to accurately call the participants trained or make any inference for how training affected results. (e.g., line 68, page 3). In addition, the investigators did not include aged-mated non trained participants. Therefore, it is suggested that all mention of training be excluded from this manuscript.

We thank the reviewer for their comments that help to improve clarity of this manuscript.

i) We have modified the description of this cohort to include “*recreationally trained runners*” throughout the text to emphasize these participants were not taken through a formal training protocol.
ii) Additionally, we have added the following text to the methods section (line 43-46), “*The average VO2max of NWT and OWT participants was measured as previously published [9]. In the OWT group, it was 62.0±2.1 (mL/kg lean mass/min), whereas in the NWT group, it was 73.3±5.3 (mL/kg lean mass/min). Participants were recruited with the goal to achieve similarity in age and sex between the two groups.*”
iii) We also acknowledge the limitation of the study, recognizing the absence of a non-trained or sedentary comparison group in the text (line 243-246): “*Our study involved recreationally trained runners, capable of continuous running for 90 minutes at 60% of their VO2max [9]. Whether these differences are unique to trained humans remains unknown and requires further study to determine how specific compartments dynamically produce and consume FAHFAs in varying states of adiposity and in specific lean and adipose tissue depots*.”
iv) Finally, we added following text summarizing the manuscript (line 253-255): “*Limitations of this study include its small sample size, lack of historical data on the training experience and diet of participants, and a lack of an age-matched nontrained cohort. The study is strengthened using a quantitative, targeted lipidomics platform to measure FAHFAs in human serum.*”
2. If in fact, these participants were regularly running, no information was provided for the time frame between the last bout of exercise and blood draw. Aerobic exercise is known to modulate circulating fatty acids. In fact, the investigators previously showed that FAHFAs were dynamically regulated during acute exercise in lean participants (line 26, page 1). Without a standardized time frame for this, the acute effect of the last bout of exercise may have influenced the circulating FAHFA values and therefore biased the results.

We appreciate reviewer’s comment. We added following text to the Method section (line 47-51): “*For baseline blood sample collection, participants were instructed to avoid intentional exercise for two days and arrived for the visit after an overnight fast (minimum 8 hours). These samples were used to measure baseline FAHFA concentrations as well as insulin and glucose levels. Insulin sensitivity was estimated by homeostatic model assessment for insulin resistance [HOMA-IR: (fasting serum insulin (uU/mL) fasting glucose (mmol/L))/22.5] [11, 12].*”

No information was provided about the current diet of the participants. As with the acute effect of exercise, this could have also influenced circulating FAHFA values and therefore biased the results.

To improve clarity of the study parameters we have expanded the methods section, adding the following text, ““*For baseline blood sample collection, participants were instructed to avoid intentional exercise for two days and arrived for the visit after an overnight fast (minimum 8 hours).*”. We also acknowledge the cross-sectional study design and lack of information on diet, which may independently influence the FAHFA lipid profile, as limitations of this study, we added following text summarizing the manuscript (line 253-255): “*Limitations of this study include its small sample size, lack of historical data on the training experience and diet of participants, and a lack of an age-matched nontrained cohort. The study is strengthened using a quantitative, targeted lipidomics platform to measure FAHFAs in human serum.*”

3. The investigators state that the clustering of FAHFA distributions by BMI support the hypothesis that excess adiposity influences regulation of FAHFA turnover. (lines 93-94, page 4). However, it has been shown that BMI is not a good measure of adiposity as some individuals who present with high values do so because of large LBM. Please consider revising.

We agree with the reviewer that BMI is not necessarily reflective of excess adiposity. We show here and in previous publications that while BMI does differentiate normal weight and overweight recreational runners, it only moderately correlates with FAHFAs. Instead, body fat shows a stronger relationship [Nelson, et al 2022, PMID: 35192550]. To emphasize this important distinction, we now demonstrate here, via univariate linear regression, that BMI alone, while statistically significant, is not a strong predictor of total FAHFAs in circulation (see new Supplemental Figure 1A). We also show that adjusting for gender improves prediction significantly (see new Figure 1B), thus motivating the analysis of the influence of increased adiposity -independent of gender - on FAHFAs (all female participants, who had similar amounts of LBM) and the influence of gender, independent of BMI, on FAHFAs (all NWT participants, who had similar amounts of total fat mass).

4. Was FAHFAs different when pooling all females versus males studied? Why did the investigators choose to only examine this relationship between NWT males and females? The investigators state that females had a higher FLR than men; infer that this anthropometric index could be driving a difference in FAHFA; but do not show how this affected FAHFAs other than matching for BMI (see previous comment on BMI). They point to the fact that when lean and overweight groups were combined, FAHFA concentrations show the expected negative association with body fay (Figure 2). However, this data was only examined in female subjects. Going back and forth between data sets only confuses the interpretation of the results.

We agree, following changes have been made to the manuscript:

i) To improve clarity of the manuscript we have added text which we think better explains rationale for the subgroups chosen for determining associations amongst body composition and circulating FAHFAs: “*Both BMI and gender are known to influence adiposity and lean mass (i.e., distribution and size) [16]. As we observed both gender and BMI-related differences in circulating FAHFAs, we hypothesized adipose depots may underlie these differences (Table 1).*”
ii) We have now included analysis of the male participants in Supplemental Figure 2a where we observed similar trends with moderately weaker Pearson correlation coefficients given the smaller difference in circulating FAHFAs compared to female participants (Figure 1A).
iii) To further support observations that FAHFA pools are influenced by lean and fat mass, which are modified by obesity, we have added an additional panel (Figure 3F) modeling total FAHFAs from the interaction between lean mass and total fat mass (R^2^ = 0.73).
4. The investigators comment on the fact that visceral adipose tissue is associated with cardiometabolic disease risk (line 47, page 2) and conclude that pathogenic adipose expansion, particularly in VAT, negatively influences circulating FAHFA concentrations. However, the investigators practically ignore to mention in their discussion (and direct analysis) the group (OWT males) who clearly had the most VAT. Information from this group needs to be added to the manuscript and the interpretation of results explained in the discussion.

We appreciate reviewer’s comment. We have added analysis of all males as well as analysis of the OWT group to Supplemental figure 2. We have also added text highlighting the negative association of VAT and FAHFAs in both the analysis of female participants (adiposity influence on FAHFAs) and the analysis of NWT (gender influence on FAHFAs)(line 202-204): “*Moreover, while VAT retained its negative association to FAHFAs, relationships to total fat mass, SAT fat (13 of 25 species, R ≥ 0.5), and gynoid fat (13 of 25 species R ≥ 0.5) were positively associated with baseline circulating FAHFA concentrations in the NWT cohort, which was also unexpected.”*

5. The sentence on lines 75-76 (page 3) is somewhat confusing and needs further explanation.

We agree and have rephrased this statement for clarity. Now it states (line 193-194): “*This unexpected divergence suggests these species might be independently regulated by hormone-fuel relationships rather than adiposity.*”

6. The number of subjects was quite low in each group and limits the interpretation of results.

We agree and note this limitation in the discussion. Following text summarizing the manuscript was added (line 253-255): “*Limitations of this study include its small sample size, lack of historical data on the training experience and diet of participants, and a lack of an age-matched nontrained cohort. The study is strengthened using a quantitative, targeted lipidomics platform to measure FAHFAs in human serum.*”

Reviewer #2: This original research manuscript entitled “Gender-based heterogeneity of FAHFAs in trained runners” provides interesting findings regarding serum FAHFA concentrations in relation to gender-based, and anthropometric-based differences in recreationally trained runners. The author group has previously published within this context (i.e., examining the acute exercise response of serum FAHFA in similar groups), and thus have experience within the niche of FAHFA serum concentrations, specifically as they relate to exercise status/capacity and anthropometrics. This article does succeed in its stated goal of determining potential differences in serum FAHFA content between gender, as well as between obese/overweight vs normal weight recreationally trained runners.

Based upon previously published work, as well as indicated by the authors, certain expected differences were observed herein. For example, results from the MS analyses revealed a strong negative correlation in much of the 25 FAHFA species in relation to total fat mass. Interestingly, the authors also pointed out that 5 certain FAHFA species were not correlated with fat mass, but were however correlated with serum glucose, insulin, and HOMA-IR values. There were some findings that the authors indicated to be unexpected; the observed negative relationship between LBM in 14 out of the 25 FAHFA species in NWT vs OWT (males & females combined) appears counter to the paradigm that leanness is positively associated with FAHFA content.

Regarding the LBM-FAHFA negative relationship, it is important to consider that obese individuals often have greater LBM compared to normal-weight counterparts. This stands to reason, as adiposity—and body mass—is accumulated specifically by a net-positive energy balance. The calorie surplus that results in an individual becoming overweight or obese is not exclusive to adiposity, rather increased skeletal muscle is also well-described. This highlights an inherent shortcoming of solely relying upon correlational data to guide future work and/or hypotheses. The authors should likely highlight that a potential explanation of this observed negative relationship between LBM and FAHFA content is that obese individuals often (and within this study itself) exhibit greater total LBM to normal-weight counterparts as a side-effect/consequence of the net positive energy balance responsible for adiposity/obesity. Overfeeding—absent of other direct stimulus (e.g., hypertrophy/resistance training)—can be a potent stimulus for mTOR activity and skeletal muscle growth. Conversely, long-term endurance training expends a significant energy and often results in a net negative energy balance and body weight loss overtime—this includes the loss of LBM. Accordingly, it’s evident that NWT exhibit reduced LBM compared their OWT counterparts. The present manuscript exhibits correlational analyses to examine specific fat mass depots, fat mass, and lean mass, independently. However, there does not appear to be any direct correlational and/or other analyses examining body composition specifically.

We thank the reviewer for these comments. We agree that LBM plays an important independent role. To emphasize this point we have included a new multiple linear regression analysis that includes lean mass, total fat mass, and their interaction as predictors of total circulating FAHFAs (new Figure 3B). These results indicate the altered ratio of lean to fat mass in obesity may alter the source/sink dynamic of FAHFA turnover.

Specific Comments:

-The authors should explicitly perform analyses to examine body fat % compared to total mass and total LBM % in relation to FAHFA content. This will better elucidate if the observed negative LBM-to-FAHFA relationship is driven by LBM content alone, OR rather a relationship driven by obesity itself (as obesity itself is associated with > LBM).

We thank the reviewer for this important observation. We have now included both percent body fat and percent lean mass in a supplemental analysis (Supplement 2A, 2B). We agree there appears to be a complex interplay between lean mass and fat mass expansion in obesity that disrupts FAHFAs. Future investigation of uptake and subsequent fates of FAHFAs by lean tissues will be necessary to elucidate how these mechanisms are modulated by obesity.

-Recent literature highlights some inherent issues associated with the type of MS approach utilized to quantify serum FAHFA. One such paper (Nelson et al., Artifactual FA dimers mimic FAHFA signals in untargeted metabolomics pipelines. J Lipid Res. 2022 May) highlighting FA dimers and artifacts in this type of metabolomics pipeline, specifically cites a previous study conducted by the current authors. However, the authors make no explicit mention of this within the manuscript as either an acknowledgement of the analytical issues inherent in the type of untargeted metabolomics pipelines used in the current manuscript.

We thank the reviewer for highlighting our own publication in the *JLR* (PMID: 35315332), which we neglected to include in the current work in the prior submission. We have now included new text in the methods and discussion emphasizing our targeted lipidomics approach and the consideration of analytical method required to further study FAHFA turnover.

i) In the Method section now it states (lines 75-80): “*Lipids were extracted using a modified Bligh and Dyer protocol and solid phase extraction with a HyperSep silica SPE cartridge at room temperature. Before infusion, FAHFAs were derivatized with N-[4-(Aminomethyl)phenyl]pyridinium (AMPP). Then, derivatized extracts were infused in TSQ triple quadrupole mass spectrometer (Thermo Fisher Scientific) equipped with an automated nanospray device (Triverse Nanomate, Advion Biosciences, Ithaca, NY). Identification and quantification were determined by product-ion analysis.*”
ii) In summarizing statement we added following text (lines 254-259): “*The study is strengthened using a quantitative, targeted lipidomics platform to measure FAHFAs in human serum. Previous work from our lab has shown that untargeted metabolomics analytical methods are susceptible to the formation of fatty acid dimers that mimic FAHFAs, thus confounding interpretation [19]. Here we use both solid phase extraction and careful identification of FAHFAs by selected ion monitoring to ensure proper identification and quantification of this lipid class and their species.*”

-The rigor and technical soundness of this manuscript would be greatly improved with the inclusion of additional/complimentary techniques/analyses. Specifically, this manuscript is almost entirely grounded in one technique (MS shotgun metabolomics), and almost entirely within the analytical context of Pearson correlations. It’s very difficult to draw any solid conclusions from omics and correlations alone. It would be my recommendation to include some form of lipid species examination in addition to the MS data, as a means of confirmation and/or as an adjunct to the present study.

We apologize for the omission of important method details in our previous submission. We now include more details on the formally quantitative lipidomics approach used in this manuscript to measure FAHFAs (please also see point above)
